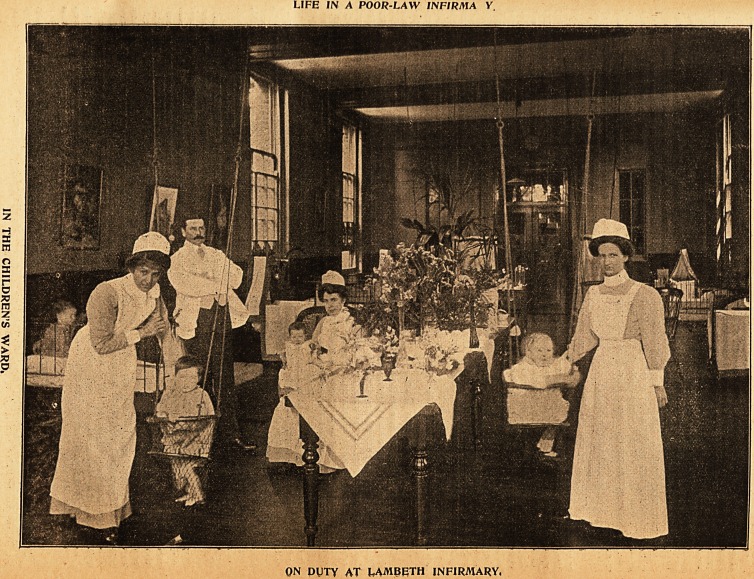# The Hospital. Nursing Section

**Published:** 1907-03-16

**Authors:** 


### The Hospital.

### Nursing Section.

Contributions for "THE HOSPITAL," should be addressed to the EDITOR, "THE HOSPITAL" NURSING SECTION, 28 & 29 Southampton Street, Strand, London, W.C.

No. 1,070.—VOL. XLI SATURDAY, MARCH 16, 1907.

### Notes on News from the Nursing World.

### THE EMPRESS MARIE AND INTERNATIONAL NURSING.

ONE reason why Queen Alexandra visited the Jubilee Institute and the London Hospital last week was that no subject is nearer to the heart of her illustrious guest and relative, the Empress Marie, than the adequate care of the sick and wounded. It will be remembered that during the war between Russia and Japan the Empress Marie was foremost in raising money for the equipment of nurses; and she was so impressed with the sufferings of the injured soldiers that she devoted, from her private purse, a sufficient sum of money to found the Empress Marie Féodorovna Fund for the purpose of awarding £2,000 in prizes for the best practical inventions for diminishing them. We have already intimated that these will be exhibited by the British Red Cross Society in June, and Her Majesty's brief stay here will tend to quicken the interest in the exhibition. With regard to nursing itself, the district nurse, as we know her, is quite unknown in Russia; and Queen Alexandra was only too pleased to show her sister how the work is managed at the headquarters of our district nurses. At the London Hospital, which was selected because it is the largest and was the first to have installed the Finsen light from Denmark, she had an opportunity of seeing how the nurses of a great institution live and work.

### ROYAL NATIONAL PENSION FUND FOR NURSES.

WE remind our readers that the annual general meeting of the members of the Pension Fund will be held at River Plate House, Finsbury Circus, on Thursday, March 21, at 4 P.M. Any policy-holder of the Fund who wishes to be present will be welcomed by the Council, and tea will be provided after the meeting. Policy-holders attending are asked to write their names and policy numbers legibly on a card to be handed in on entering.

### THE JEWISH GUARDIANS AND THE EAST LONDON NURSING SOCIETY.

MENTION is made in the annual report of the East London Nursing Society, whose annual meeting took place at the Mansion House on Tuesday afternoon, of the addition which, thanks to the liberality of the Jewish Board of Guardians, has been made to the staff. Early in the year the Board appointed a Special Committee to inquire into the provision by existing agencies for nursing the Jewish sick poor in the East end. After investigation the Committee advised their Board to recognise the active labours of the Society among the Jewish East-end poor by offering to subsidise an additional nurse. This has since been done, the Board agreeing to pay a sum of £80 a year for the nurse, who, if possible, is to belong to the Jewish race, preference being given to one speaking Yiddish or Jewish. The arrangement thus entered into is said to be working well. The Society also benefited last year, for the first time, by a grant of £150 from the Hospital Sunday Fund; the Royal Amateur Art Society contributed £185; a sale of Lady Maxwell Lyte's drawings realised £57; and the proceeds of a concert at Bechstein Hall £45. The income of the Society, in fact, exceeded the expenditure by about £100, but the latter included a legacy of £200 from the late Miss Alice Phillimore, for fifteen years an active member of the Ladies' Committee. The work for which Lord Crewe, Archdeacon Sinclair, and others pleaded at the meeting goes on steadily. During the last twelve months the staff of three matrons and twenty-five nurses paid 104,500 visits to 4,639 patients, and an average of 4,180 visits were paid for each nurse employed. We notice with pleasure that the Committee return warm thanks to the nurses for their zeal and efficiency.

### GUARDIANS AND TITTLE-TATTLE.

THERE has been an angry discussion at a meeting of the Keighley Guardians in consequence of assertions by some member of the Board that the nurses are afraid of the matron. A letter repudiating this statement was signed by eight of the nurses, who said: "We do get on very well with the matron, and we must say that when tales to the contrary come to the Guardians indirectly, as they admit, they had evidently grown in the telling. We are not able to trace amongst ourselves any dissatisfaction in connection with the treatment meted out to us by the matron." They stigmatised as false a particular allegation that "the nurses dare not speak and dare not call their souls their own." The medical officer also defended the action of the matron, and it appears to us that the discussion only affords yet another illustration of the folly of individual Guardians listening to tittle-tattle respecting the staff of institutions which are under their management.

### LORD SHAFTESBURY ON DISTRICT NURSING.

IT is pleasant to see the owner of the honoured name of Shaftesbury taking a practical interest in the nursing movement. The present Lord Mayor of Belfast is Lord Shaftesbury, and in moving the adoption of the report of the Society for Providing

Nurses for the Sick Poor, he maintained that there is no charity in the city which has a greater claim on the liberal support of the public. He dwelt on the services rendered by district nurses to the patients discharged from hospitals as incurable; on the advantage to the poor in having the advice of a nurse who understands the principles of hygiene and cleanliness, never more needed than at a time when the disease popularly known as spotted fever is rife in Belfast; and on the service performed to the community by the prompt manner in which the nurses give notice of cases of tuberculosis and cancer to the health authorities. Lord Shaftesbury's admirable plea will, we hope, be so taken to heart by the citizens of Belfast that when the next annual meeting is held the deficit of £85 will have been wiped out.

### DISMISSAL FOR TEMPER.

An asylum nurse was charged the other day with an assault on a patient in the County Asylum at Lancaster. The Director of Prosecutions instituted the proceedings, and it was stated that, while attempting to brush a patient's hair, the nurse was attacked, and, subsequently, losing her temper, struck the patient twice with a slipper. Another nurse in the institution said that the defendant struck the patient with a felt slipper, but only in order to release herself from the grasp of the latter, who was violent and had attacked other members of the staff. The medical superintendent gave evidence to the effect that the nurse admitted to him that she had done wrong, and expressed her sorrow. He complimented her on telling the truth, and dismissed her on the spot. The Bench then dismissed the charge, and, we think, very properly. Of course a person who cannot control her temper is not fit for a mental nurse, because she is sure to be provoked, but the dismissal without notice was, in this case, a quite sufficient punishment.

### THE DOCTOR AND THE NURSE'S FEE.

In the course of evidence given on Monday in a case tried at Brompton County Court the plaintiff, a medical man, who sued another medical man for a sum due to him as an anæsthetist, was asked by counsel for the defendant whether a doctor employing an anæsthetist was liable for his fee, providing the patient did not pay him. Receiving an answer in the affirmative, he went on to inquire whether, when a doctor recommends a nurse, he would also be considered responsible for her fee in the event of the patient not being able to pay it. The reply was "Certainly, if the patient had not paid her." Nurses who are recommended by medical ment to patients will make a note of this statement.

### THE PARIS PICNIC: NEITHER INTERNATIONAL NOR PROFESSIONAL.

It will be noted from an advertisement in another column that we have republished the articles on "Make-Believe in British Nursing," and that they may be obtained separately in pamphlet form. Many of those who have the best interests of British nursing at heart will welcome their republication in a form which will make them readily available for constant reference. That these articles have had some effect is clear from the latest official announcement of the arrangements for the Paris Picnic. The promoters have now publicly admitted by the decision they have come to that there will, in fact, be no International Conference of Nurses, as was originally announced. This is clear from the statement that no official delegates will be invited to Paris. The meetings are to be thrown open to members of the lay public who wish to give or gain information, as well as to nurses and members of the medical profession. It will be seen, therefore, that the meetings will be admittedly non-professional and non-international. We look forward with great satisfaction to the discussion, in which the lay public is invited to take part, "On Professional Organisation; the History of the Professional Nursing Press."

### FROM BIRKENHEAD TO LONDON.

Miss Cockrell, who has just been elected matron of St. Marylebone Infirmary, makes the third head of important Poor-law training schools in London whose previous position has been that of lady superintendent of Birkenhead Union Infirmary. The medical officer at Birkenhead, who may be congratulated on the fact that there are now twenty-nine nurses for 400 beds, as against four nurses for 360 beds eleven years ago, and the Guardians naturally regret the removal of Miss Cockrell; but it may be hoped that her successor will prove equally capable.

### INTERESTING BEQUEST TO QUEEN'S NURSES.

The late Miss Guthrie Wright, of Edinburgh, has left the residue of her means and estate to the Scottish branch of the Queen's Institute for Nurses. According to her will, £1,000 of the amount is to be devoted towards making provision for superannuated or disabled Queen's nurses, either by pensions or by providing houses, as the Council may in their discretion see fit. The sum of £500 is to go to the Nurses' Sick Fund, the interest only being used; £500 is to be set apart, and the income used for providing drives for patients and nurses; and £1,000 is to be employed for the endowment of an existing or new district in Edinburgh. If there is any balance of residue, it is to be appropriated for the general purposes of the Institute or for any specific purposes which the Council may deem advisable.

### BICYCLES FOR NURSES.

The report of the Carlisle and District Nursing Association, which was read at the annual meeting the other day, shows that the committee were just able to make things square, the balance of increase over expenditure being five shillings. But the chairman, in his speech, stated that another £50, increasing the income from £450 to £500, is needed. Warm appreciation of the work of the matron, Miss Little, and her staff of five nurses was expressed, and Dean Ridgeway, whose experience in London is sure to be useful to him in Carlisle, said he was glad to notice that the poor themselves are grateful for the care and attention afforded to them. Last year an appeal was made by the committee for bicycles, and the response was most gratifying, four useful machines—one wholly new—having been sent. The distances traversed by the nurses is very considerable, and they are able, by the aid of the bicycles, to get through much more work with less exertion.

### CHILDREN'S NURSES IN AUSTRALIA.

An important new departure has been taken by the Committee of the Foundling Hospital, Melbourne, who have decided to engage in the work of training children's nurses. Lessons are to be given to the trainees in the preparation and administration of food to children of all sorts and conditions; in the nursing of them when they are sick; and in bringing them up to habits of health and cleanliness. A certificate of efficiency will be awarded after examination. The matron is a trained nurse, who was sister at the Children's Hospital in Melbourne. She has under her a sister and a night sister, who will, in turn, instruct the pupil nurses; and special teachers of dress-making and hair-dressing will give lessons. The entrance fee is to be three guineas, and the period of training is for a year. After the first six months a small salary will be paid, and uniform will be provided by the hospital. The object in view is to attract girls of education and refinement to the work.

### DISTRICT NURSING IN BIRMINGHAM.

The Committee of the Birmingham District Nursing Society have decided to erect a new home for the accommodation of their eighteen nurses. The Lord Mayor, who proposed the adoption of the report at the annual meeting in the Council House in the name of the city, thanked all the workers for their self-sacrificing devotion, and said that the Society is one of those unostentatious but exceedingly valuable institutions in which Birmingham is so rich. Last year the nurses from Newhall Street paid more than 42,000 visits, the nurses from the Moseley Road branch 32,000, and upwards of 7,000 cases were treated in schools. The new building is to cost about £4,000, and an annual additional outlay of at least £65 will be necessary. As there was a deficiency of £77 last year, a considerable increase in the subscriptions is obviously required.

### A NURSE'S TEMPTATION.

Another sad illustration of the want of business methods shown by the average nurse is given in a case which was tried in one of the Courts last week. The charge was that the defendant, a fully-qualified nurse, who had been recommended by a Harley Street physician for fifteen years, had stolen silver-plate and other goods, value £20. The accused took a furnished house at three guineas a week, and received under her care a mentally irresponsible patient. Subsequently, to tide over temporary difficulties and to obtain absolute necessities, she pledged some of the prosecutrix's property. When the officer came to arrest her, he found her concealed under a bed in the caretaker's room. It was stated by the nurse, who was not represented by counsel, that she intended to restore the property, and that since her arrest she had redeemed some of the articles and sent the prosecutrix £5. Whilst the judge was summing up the accused fainted, and upon her recovery apologised to the judge, saying: "I have had nothing but dry bread for twenty-three days, and am not very strong." She then burst into tears. The jury acquitted her, but the judge warned her that she had been guilty of a grave impropriety. This incident would not have happened if the nurse had not been imprudent enough to start on her own account without capital.

### WORKING MEN AND DISTRICT NURSING.

A feature of the report which was read and adopted at the annual meeting of the Willenhall District Nursing Association was the substantial increase in the contributions of the working men in response to the special appeal made to them in the report of the previous year. The police and tramway men gave a donation of £32 12s. 9d., and the Midland Railway and Albion employés £13 12s. 1d., the proceeds of football matches. The sum of £20 0s. 3d. was also raised by a Saturday street collection. In spite of the additional support given, one of the speakers said that not more than a fifth of the works in Willenhall were contributing to the Association. Its financial position is, however, fairly satisfactory, and a general belief was expressed that the more its needs were known, the greater would be the assistance given.

### A GOOD RECORD AT SOUTHAMPTON.

It is not only as a port that Southampton is making progress. The branch of the Queen's Jubilee Institute is growing in importance. The nurses were able last year to move into more commodious quarters, as the result of the allotment to the Institute by the Charity Commissioners of a portion of the funds of St. Mary's Cottage Hospital, which has been closed, and there is every prospect of it becoming an increased power for good in the town as the population increases. The weak point is the number of annual subscriptions which, in the estimation of the Committee, are not so numerous as they should be. It is noteworthy that since the issue of the last report the whole of the *personnel* of the staff has changed, but the character of the work has in every respect been maintained.

### SHORT ITEMS.

A meeting of the Nurses' Social Union was held at Taunton on March 6 at the District Nurses' Home, when Dr. Rutherford gave an instructive lecture on skin diseases, illustrated by Crocker's prints; and almost every nurse in Taunton and for many miles around were present.—The names of Nurse Waldren and Nurse Lawrence, who were trained by the Kingswood and District Nursing Association, near Bristol, should be added to the list of candidates who successfully passed the last examination of the Central Midwives Board.—The resignation of Miss L. A. Wing as superintendent of the Coventry District Nursing Association was announced at the recent annual meeting, and great regret was expressed at her retirement after twenty-three years of devoted service.

### The Nursing Outlook.

"From magnanimity, all fears above;
From nobler recompense, above applause,
Which owes to man's short outlook all its charm."

### QUEEN ALEXANDRA.

THE voluntary hospitals of this country owe more probably to the members of the royal family of England than many of them quite realise. The King has shown his own belief in the privilege of personal service in the cause of the sick, on many occasions. His Majesty has permanently enshrined these principles in the King's Hospital Fund and the League of Mercy, which he has established. The King's example has been energetically followed by the Prince of Wales, the Princess of Wales, the Princess Victoria of Schleswig-Holstein, and indeed by practically every member of the royal family.

Her Majesty Queen Alexandra, whose kindness of heart is proverbial, has struck out a line of her own. She has shown her sympathy with sick people as individuals, and has identified herself, personally, to the fullest possible extent with the life of the working nurse, and indeed with that of all who spend their days in ministering to the sick. During the last week she has found time to examine the organisation of the Queen's nurses by visiting the office in Victoria Street, and meeting there the superintendents and representative nurses. On Saturday, accompanied by her sister the Empress Marie, Queen Alexandra went to Whitechapel and spent three hours in the London Hospital of which she is President. Her Majesty's was no formal inspection, but a thoroughly business visit. The Queen took pains, with her sister the Empress, to speak to every patient in every ward which she entered, and to see everything there was to see, so that she might judge for herself of the work and of its value to the suffering poor.

There are a few points which every visitor should bear in mind when inspecting a hospital. There is a twofold duty on such occasions, one to the patients in the wards, and the other to estimate the quality of the work done by an exercise of judgment and thoroughness. Her Majesty Queen Alexandra's deep sympathy with the workers and those in their charge was markedly exhibited in the course of her inspection. Not only was she careful to avoid wounding the susceptibilities of any of the patients by omitting to speak to a single sufferer, in any ward visited, but she declined to decide on the merits of particular nurses' work. Again, could anything be more graciously tactful than the Queen's expressed wish that the sisters and nurses should attend her at luncheon? We emphasise these points because we know that in visiting a hospital some great people are apt, from nervousness or a want of tact, to diminish the good which the sympathy of their presence would otherwise bring.

Queen Alexandra admirably fulfilled the duties of President by observing the two main principles we have indicated. We have shown how tender was Her Majesty's solicitude for the patients, how infinite her courtesy, how great her care, that not even the humblest and weakest of the patients should be excluded from the sphere of her gracious sympathy and personal interest. The Queen exhibited keen powers of observation and a thoroughness of desire to see everything. In the nurses' home she entered the bedrooms at random and would not confine her inspection to those prepared for her notice. Then, too, the Royal President did not wish to pass the entrance to the out-patients' department, but desired to go into the hall amongst the hundreds of patients who were there assembled. In inspections of other hospitals Queen Alexandra has shown the same thoroughness of observation, and it was mainly due to an inspection she made when Princess of Wales, of the Hospital at Netley, that that establishment was renovated, purified, and brought into a state of relative efficiency, which it had previously conspicuously lacked.

Apart from its value to the London Hospital, Queen Alexandra's visit to Whitechapel should tend to stimulate the interest of many influential people in hospital work. We believe that many will be moved, not only to visit hospitals, but to make their visits thorough and helpful to the fullest possible extent. How much nurses owe to Queen Alexandra it would be difficult to over-estimate. In the early days of the Royal National Pension Fund for Nurses, Queen Alexandra gave the strongest possible evidence of her sympathy with nurses in their work, by associating herself with the Fund as its President. This important action had the support of His Majesty, who became patron of the Fund at the same time. The King and Queen might have hesitated to undertake so grave a responsibility, especially in the early days of the Pension Fund, for it had to be conducted as a great business enterprise. Queen Alexandra, by becoming its President, did the nurses throughout the Empire an inestimable service. Her Majesty may well be proud to find her judgment so completely upheld, for the Royal National Pension Fund is, to-day, the greatest thrift organisation amongst women workers in the world. Queen Alexandra, when Princess of Wales, was lovingly known as The Princess of Pity. Her Majesty's life in these islands, during the many years she has resided amongst us, has won the hearts of the people. She is indeed a Queen of Queens, whose example has influenced the whole nation for good in many ways. Few, if any, men have ever been able to exercise a similar beneficent influence over a whole people.

### The Care and Nursing of the Insane.

By Percy J. Baily, M.B., C.M.Edin., Medical Superintendent of Hanwell Asylum.

### II.—NURSING THE SICK.

(*Continued from page* 323.)

*Charcoal Poultices* are sometimes used when dealing with very septic or sloughing and foul-smelling wounds, especially for hastening the separation of the slough in large chronic bed-sores. The charcoal absorbs to some extent the very foul odour which comes from these wounds. For a poultice which would require about half a pint of water, half an ounce of powdered wood charcoal should be used. Half the charcoal may be mixed with the linseed, the remaining portion should be sprinkled over the surface of the poultice when it is made. For such poultices boiling boracic lotion may be used instead of boiling water. Charcoal poultices are necessarily very dirty applications, and for this reason their use is not very general, but in dealing with large sloughing bed-sores they are undoubtedly most useful. From the nature of their uses they must, of course, be changed much more frequently than an ordinary linseed poultice.

*Fomentations* consist of some sort of absorbent material (flannel, sponges, spongiopilin) wrung out of boiling water. Their uses are precisely those of poultices over which they have the advantage of being cleaner and lighter, but the disadvantage of not retaining the heat so well. They should be made in a wash-hand basin which must be warmed. A towel should be placed over the basin, and on this the flannel or other material which is to compose the fomentation is laid. If flannel is used several layers will be required. The boiling water is then to be poured into the basin over the flannel and the towel, and when the flannel is thoroughly soaked it can be wrung by means of the towel. The fomentation should be wrung as dry as possible; it should be shaken out and applied as hot as the patient can bear it. Equal care, however, must be exercised here as with poultices in order to avoid burning the patient. The fomentation when it is applied should be covered with jaconet and a layer of cotton wool, and can then be fixed with bandages or binders. Fomentations require to be changed more often than poultices, generally about every two hours.

Various medicinal substances may be sprinkled over the fomentation in order to enhance its effect, such as tincture of opium (laudanum), belladonna, or spirits of turpentine. The term stupe is sometimes exclusively applied to this class of fomentation.

Unhealthy or septic (putrid) wounds are frequently dressed with hot antiseptic fomentations, which are usually made with boracic lotion.

### 8. Baths.

Water baths are used either for cleansing purposes or to obtain some special effect upon the tissues or organs of the body in disease. They are given at various temperatures from near the freezing-point up to 110° or 112°. The temperature of the water must in every case be ascertained by means of a thermometer.

For ordinary cleansing purposes the temperature of the water should be about the same as the blood—that is to say, somewhere between 95° and 100°. Every patient who receives a bath should have fresh clean water, and when the ablution is completed the bath should be thoroughly rinsed out so as to remove all soap suds from its side before being filled up for the next patient. When filling the bath the cold water must always be turned on first, and the hot water tap should never be turned while the patient is in the bath. In dealing with insane patients every care must be exercised in bathing them so as to avoid giving them any ground for charges of cruelty; many such patients have a rooted objection to the bath, and refuse to wash themselves, and afterwards make all sorts of charges against the nurses and attendants for which there should be no foundation. In washing the face care must be taken that no soap gets into the eyes or mouth, and on no account must a patient's head ever be pushed down under the water. No insane patient must ever be left alone in the bath. When the bath is finished each patient must be provided with one or more clean and separate towels.

Cold baths are sometimes used in order to reduce the patient's temperature in cases of high fever. The temperature of a cold bath may be anything below 65°. The patient is, however, rarely placed in such cold water to start with, but is put into a bath of about 85° or 90°, and the temperature is then lowered by the addition of ice or cold water. In such diseases as acute rheumatism, pneumonia, and typhoid fever the temperature of the patient may rise as high as 106° or 107°—a condition known as hyperpyrexia. Such a temperature is incompatible with life, and must be speedily reduced, otherwise the patient will almost certainly die. The quickest and surest way of reducing the temperature is to place the patient in a cold bath. Moreover, the cold bath seems to have some influence in counteracting the effects of or removing the poison of the disease from the system. Such baths are always given under the immediate direction and in the presence of the medical officer. This form of bath is resorted to only when the patient is very ill, and therefore the very greatest care must be exercised in administering it. The bath should be arranged beside the patient's bed. The patient is to be stripped and placed on a strong sheet which will bear the weight of his body without fear of tearing. A towel should be wrapped around the patient's pelvis, and he should then be covered with a blanket. He is to be lifted off the bed by means of the sheet and gently lowered into the water, the blanket which covers him remaining stretched over the sides of the bath and not reaching the water. If there are enough assistants (three or four persons at least are necessary) it is more convenient to place the bath at the foot of the bed in place of beside it, the lifters then being arranged on either side of the patient merely pass him along and over the foot of the bed, and thus save reaching and bending over him as must be the case when lifting him from the bed sideways. While the patient is in the bath his temperature must be constantly taken, either in the mouth or in the rectum. He must be removed from the bath as soon as the temperature falls to 101° or 100°, for it will continue to fall after he is taken out of the water. He must also be taken out immediately if there is any sign of collapse or failure of the pulse, or if he should shiver, or if the surface of the body becomes at all blue. When taken out of the bath he should be lifted directly on to the bed, which should now be protected with a mackintosh sheet with a blanket spread over it. The wet sheet and the mackintosh must then be removed, as well as the towel around the patient's pelvis, and his body rapidly dried and (or without drying) wrapped in a warm blanket. If there is any sign of collapse or shivering, hot water bottles to the extremities may be necessary, and stimulants in the shape of hot brandy and water may be given. In the case of women the bath should not be administered during menstruation.

In healthy people the general effect of the cold bath is at first depressing. The vessels of the skin contract, the blood being driven to internal organs. The pulse becomes slower by 10 or 20 beats per minute. This is followed by reaction in about four or five minutes, when the bath should cease, and be followed by vigorous rubbing of the skin with rough dry towels. Cold baths tend to increase the appetite.

Hot baths vary in temperature from 100° to 112°. Their effect upon the skin is to enormously increase the amount of blood circulating in it by dilating the smaller arteries. They also stimulate the sweat glands, producing profuse perspiration. The action of the heart and the pulse-rate are accelerated. They are a most useful means of relieving pain and muscular spasm.

*Hot Air and Vapour Baths* are sometimes used. They generally require some sort of special apparatus for their production, and their description does not appear to fall within the scope of this work. A vapour bath may be improvised by placing hot water bottles or hot bricks covered with wet stockings or wrapped up in wet flannel in the patient's bed. They should be placed upon folded blankets to protect the under bed-clothes and covered with cradles so that the upper bed-clothes may be kept off them. During their use the patient should lie between blankets. This sort of vapour bath is useful to produce sweating, especially in kidney disease.

### The Care of the Throat, Nose, and Ears in Children.

By St. CLAIR THOMSON, M.D., F.R.C.P.Lond., F.R.C.S.Eng., Physician for Diseases of the Throat in King's College Hospital.

"*The loss which the Commonwealth suffers by the destruction of its youth is like the loss which the year suffers by the destruction of spring.*"—Pericles.

As we are all aware, the struggle for life not only exists between races, nations, animals, and individuals, but a continual struggle for life is going on in the organism of each one of us. Life consists to a great extent of an adaptation of internal defences to external circumstances. It is by studying nature's own methods of defence that we will be able to indicate the care required for the nose, throat, and ear in children. You may remember that some frivolous person has remarked that illness was like a struggle between two people, and that the doctor resembled the third man who intervened to separate them with a club. Sometimes he hit the disease on the head and sometimes the patient! Napoleon the Great, with a flash of genius, once remarked to his physician, "Believe me, we had better leave off all these remedies; life is a fortress which neither you nor I know anything about. Why throw obstacles in its defence?" But a hundred years have elapsed since Napoleon made this pregnant remark, and in the last century medical, surgical, and sanitary science has made more progress than in all the preceding centuries of the Christian era put together. Since Napoleon's day we have learned a great deal about this earthly fortress in which life is temporarily entrenched; we recognise most of the enemies trying to undermine it; and we know most of the methods by which it is able to keep the flag still flying.

Of all the defensive mechanisms of the body few are equal in importance to that of the nose. The chief functions of the nose are as an organ of smell, as a resonating chamber for the voice, and as a filter for the air we breathe. The nose is the natural and sufficient channel for breathing through. The mouth should only be used as an accessory air-way in case of emergency. This nasal air-way has no rest day or night. Many infections are introduced into the throat by the open mouth, or into the stomach by the food; but these doors of entry are closed all night, and are only occasionally open during the day. At night the eyes are closed, the ears are insensitive, the brain is at rest, and the heart beats are slackened; but breathing goes on steadily and unconsciously, and every breath is purified, and noxious germs are filtered off from it, all night as all day.

A glance at a model will show the convoluted lining of the nasal cavities. This lining is convoluted so as to expose a large surface to the air. It is continually bathed with sticky mucus; and it is on this sticky mucus that germs are emmeshed, and here they get fixed, much as bird-lime will capture a little bird. Not only do the germs passing into the nose get stuck on this surface, but they are quickly turned out by what we know as ciliated epithelium. The surface of the delicate lining of the nose is covered with millions of minute microscopic rods. All through life these keep waving, like the tentacles of a jelly-fish, and they keep waving always in one direction—towards the orifice of the nose. When this is viewed under the microscope it looks like a field of wheat waving constantly in one direction. Any irritating particle of dust which gets into the nose is often ex-

**THE CARE OF THE THROAT, NOSE, AND EARS IN CHILDREN**—*continued.*

pelled more quickly by the abundant flow of mucus which it excites. But the minute germs which try to invade our nasal fortress are quickly passed down by these waving rods ("cilia," as we call them) to the entrance of the nostrils. The entrance of the nostrils is further protected by hairs. During a foggy day in London, or a dusty railway journey, we have only to wipe out the entrance of the nose and see the amount of dust which these hairs will arrest. The microbes, then, are wafted down to this same region, and as they are entangled in the mucus they cannot do any more harm. To realise how much work the nose has to do in this purifying process it is well to recollect that on an average in twenty-four hours we inhale 10,000 litres of air. At the lowest estimate 1,500 organisms are inhaled into the nose every hour, and it must be a common event, at least in our average London atmosphere, for 14,000 organisms to pass into the nose during an hour's tranquil respiration. This would give 336,000 microbes for the nose to deal with as one day's work. I have not yet calculated the number with which the nose has to struggle when walking behind a lady with trailing skirts, travelling in a twopenny tube, or trying to jump on a motor bus! The lining membrane of the nose has other functions. No matter what the temperature of the outside air may be, the nose is sufficient to warm it comfortably before it reaches the throat; the nose also, if the air is too dry, moistens it to a suitable degree.

Thus, before the air gets near the lungs it is filtered of dust and microbes, and agreeably warmed so as to make it fit for respiration. From the back of the nose a tube passes up to the ear, so as to supply an equality of air pressure on each side of the drum. When this tube is blocked, as it is when one has a cold, we all recognise the interference with acute hearing.

But behind this first line of defence there is another line of defence in the throat. We all know what and where the tonsils are. Besides the two tonsils which we can see at the back of the mouth there is another tonsil at the back of the nose. This latter tonsil does not make its presence known until it gets diseased or too much enlarged, when it is recognised as an adenoid growth. What exactly is the duty of the tonsils is not completely understood; but there can be no doubt that, as they are absent in the healthy adult and always present to some extent in childhood, they serve to protect the body from its outside enemies. There are other defensive arrangements, such as sneezing and coughing, which all help to keep the fortress intact.

Now we can understand the importance of the upper air passages in the preservation of health. We are now able to explain how they act, although, empirically, it had long been recognised that it was more natural to breathe through the nose than through the mouth. An American wittily remarked that "Man was the only animal that breathed through his mouth, and he was a fool when he did so." Many years ago a layman wrote a small book with the engaging title "Shut your Mouth and Save your Life." While in the most sacred of books we read that "the breath of life was breathed into man's nostrils."

Just as the mouth masticates and prepares the food for the stomach, so does the nose prepare the air for the lungs. When the nose is premanently obstructed, mouth breathing has to be adopted. Life can still be carried on—for Nature is full of compensations—but at a disadvantage. The cold air, unmoistened and unfiltered, enters through the mouth, drying the throat and exposing the lungs to greater danger. The teeth are more apt to decay; and they do not grow properly. Tennyson has recognised this in Locksley Hall, where he refers to an individual as "He with the rabbit mouth ever agape"; and George Herbert long ago wrote, "Look to thy mouth, diseases enter there." When the nose is not used for breathing purposes it, like other unused things in life, ceases to develop properly; and although the mouth serves to replace it, it only does so imperfectly.

(*To be concluded.*)

### Incidents in a Nurse's Life.

### FIRE!

It was 9.30 A.M., and I had just made my patient comfortable for the day, when I was suddenly told that the house next door was on fire, and that our rafters had already caught. The doctor (who was on the sick list himself) had sent his son to say that the patient must be moved at once. She is 85 and completely paralysed, so the task was no light one. A neighbour offered to receive her into her drawing-room, as the fire was most serious, the buildings being old, and seven fire brigades required to cope with the flames.

I first locked the front door to prevent the rush of excited people offering assistance, then I rolled the patient in a blanket, and together with the doctor's son and the housekeeper, carried her down the long strip of back garden across the road, and laid her quietly on the sofa in the lady's drawing-room. I feared the removal might upset her, as she has been bed-ridden for so many years, but after taking some nourishment she seemed as usual, and by two o'clock, to my great joy, the flag went up to indicate that the fire was extinguished. The doctor's locum was coming at three to help us back, but as the crowds had now dispersed I realised that my chance of moving my patient quietly had come. So I quickly turned her bath-chair into an efficient ambulance, by putting a box at the foot, and using a small mattress, and again having the help of the housekeeper, I laid her on it and wheeled her back, carrying her gently upstairs and placed her once more on her comfortable bed, none the worse for the experience, and apparently unconscious that anything unusual had happened. Upon the doctor's arrival at three, he was agreeably surprised at the prompt removal, exclaiming, "Well, nurse! you have done well!" Imagine my astonishment next morning when an old parishioner called, greatly distressed, having heard that Miss —— had been burnt in her bed. Smilingly I told him that she was as comfortable as usual.

### Illustrations of the Life of a Modern Nurse.

LIFE IN A POOR-LAW INFIRMA Y.

IN THE CHILDREN'S WARD.

ON DUTY AT LAMBETH INFIRMARY.

### The Queen and the Empress Marie as Hospital Visitors.

### A PRESIDENTIAL INSPECTION.

On Saturday morning the Queen and her sister the Empress Marie spent over three hours in the London Hospital. Realising that in such a large and busy institution anything in the nature of a surprise visit might mean a serious disarrangement in the work of the hospital, her Majesty signified her intention by means of the telephone on Friday evening, and in response to an inquiry from the Chairman intimated her willingness early on Saturday morning to lunch at the hospital. Shortly after noon the Royal party arrived in three carriages, being received by Lord Stanley, Treasurer, the Hon Sydney Holland, Chairman, Mr. E. Morris, Secretary, and Miss Eva Lückes, Matron.

### In the Nurses' Home.

The first visit paid was to the Nurses' Home, opened two years ago and named after the present matron. Here the Queen was shown into the sitting-room, where she spoke to several of the occupants—the night nurses had been allowed to remain up for the occasion—asking one if they often availed themselves of the piano to get up concerts amongst themselves, and laughing heartily when her attention was called to the "cap-straightener," the name given to a looking-glass placed just inside the door, so that nurses may give a last look to see if they are tidy before returning to the wards. At this point the Danish sister, Miss Larson, was presented to their Majesties, who welcomed her most kindly, and requested her to accompany them throughout their inspection.

### In the Cookery Room.

The lift was next taken to a room on the third floor, where 40 or 50 nurses were busy with sick-room cookery, making blancmanges, custards, and beef-tea. Both the Queen and her sister tasted several of the dishes, and the former seemed especially pleased to notice a milk jelly, which she pointed out to the Empress as being one of the dishes the King had been allowed in his early convalescence in 1902, when it will be remembered that he was attended by a nurse from the London Hospital. About a dozen nurses were arranging "sick men's trays," similar to those exhibited at the last Cookery Exhibition at Westminster. Some were decorated with violets, others with daffodils, and in each a certain scheme of colour was worked out. The Queen was asked to decide which she thought the best, but she only smiled and said "they were all so pretty that it was quite impossible to say."

### Inspecting the Bedrooms.

Upon leaving this room it had been proposed that the Queen and the Empress should be shown the first few bedrooms along each corridor; and with the object of making these rooms particularly pretty their occupants had early repaired to Covent Garden to buy flowers. But somewhat to the amusement of her cicerones, the Queen took the matter into her own hands, strolled up the passages, opening a door here, and peeping into a room there, as she felt inclined, so that the nurses who least expected to be honoured ultimately learnt that the Queen had visited their own particular bedroom.

### The Queen and the Children.

As the Queen and the Empress crossed the large quadrangle at the back on their way to the hospital proper every balcony was crowded with patients, nurses, and attendants, and about six hundred nurses were drawn up in the square itself, together with a large number of students. The first ward visited was that occupied by the children, where every little inmate was spoken to, one litttle boy from Norfolk especially pleasing the Queen by his country salute. Another child, on noticing that the Queen and some of the suite had gone on the balcony and the Empress was looking round as if wondering where they were, called out, "Yer can get out that way," and was thanked with a gracious smile. The maternity ward with its nine mothers and seven babies came next on the list, every mother having a few words addressed to her. Next luncheon was served in the Committee Room, the Queen requesting all who had accompanied her round the hospital to join her.

### Sisters as Waitresses.

The Royal footmen had been bidden to render any assistance desirable, and in many little ways they made themselves useful beforehand; but when the Queen sat down to table she at once dispensed with their services, and the sisters waited on the guests, Sister Larson attending in particular on Queen Alexandra. The luncheon party was a merry one, and much diversion was caused when her Majesty rallied one of the sisters. It was almost an hour before the tour round the hospital was resumed, and owing to a change in the arrangements the Royal visitors passed unexpectedly through the men's accident ward, saying a few words to all the fifty-one patients. The Queen in particular noticed a man suffering from a fractured leg, and, placing her hand near the heel, said, "That is where the pain is worst, is it not?" Upon the patient acquiescing, the Queen remarked, "Yes, I remember that well when my leg was bad," and, turning to the nurse, she added, "I found that if the leg was slung from a cradle sometimes the support eased the pain; perhaps it would help this poor fellow."

### The Empress and the Out-Patients.

The lupus room and the Finsen light were not forgotten, and here the Queen congratulated one young woman who had been successfully treated upon her "beautiful complexion," and spoke to several others, and also entered the gallery which overlooks the out-patient department. Both she and the Empress laughed considerably when they learnt that the attendance was a record one for a Saturday, and that several patients who need not have come for treatment till the following week had grown suddenly so anxious about their ailments, when it was rumoured that the Queen was to visit the hospital that day, that they had been compelled to come to be seen to at once. The Empress Marie naïvely remarked, "I wonder what they'll tell the doctor?"

Shortly after three the programme arranged had been worked through, but the Queen still asked if there was anything more they could see, and was taken to the ophthalmic department. To reach this the Secretary had to unlock a door, and as he sought to fit the key the Queen said apologetically, "This is all my fault," observing to the Empress afterwards, "I am sure they will be glad to get rid of us now."

Before leaving their Majesties signed the Visitors' Book, and expressed themselves as exceedingly pleased and interested in all that they had seen.

### Lord Crewe on District Nursing in East London.

The annual meeting of the East London Nursing Society took place on Tuesday last at the Mansion House, under the presidency of the Lord Mayor. Among those present were Lord Crewe, the Duchess of Montrose, Lady Graham, Miss Ogilvy, Mr. John Tennant, and Archdeacon Sinclair.

The Lord Mayor, in opening the meeting, said how glad he was to welcome such a Society to the Mansion House, for it was a Society which recognised no distinction of Church or sect, and with whom it was "not a question of creed, but of need." It was, however, impossible to make the work as useful and effective as it might be owing to limited funds, and he hoped this meeting might influence more people to become subscribers.

Archdeacon Sinclair, in proposing that the report (of which the Secretary had read extracts) and balance-sheet be adopted, said he was very pleased to be able to speak on behalf of the organisation, as in the districts of North and East London, over which he was archdeacon, he was only too painfully aware of the miserable conditions which this Society did so much to alleviate.

Lord Crewe, Lord President of the Council, proposed that "this meeting, having heard an account of the work carried on by the East London Nursing Society for the past 39 years, commends it to the liberal support of the public." He observed that in his work at the Privy Council he was brought into frequent contact with the nursing profession, and had had the pleasure of seeing something of those who were in it, and consequently he felt it was a privilege for him to say a few words in support of that branch of nursing which was by no means the least important. He for one could never think, without astonishment and great thankfulness, of the manner in which the nursing of the sick had developed. Only 63 years ago "Martin Chuzzlewit" was published, and though it might be called a satire or a caricature, yet it represented to a certain extent the manners of the day, and now the two famous characters of that book were as unknown in the nursing profession and as remote from to-day as Don Quixote. With regard to the special work of this Society, it supplied a particular want. There was a magnificent network of hospitals in London, but these were unable to cope with every class of need. There were the incurables, and those who might not be able to attend as out-patients and yet could not enter the hospitals. To these the district nurse was invaluable, and he was sure also that an increase in general orderliness and cleanliness in the home and in the quality of the cooking often dated from a nurse's visit. There were two things which struck him in this Society. One was that the training of the nurses was of a high standard, and the other that its work did not overlap with that of any other society, which was so often the case with many institutions which the public were asked to support. He felt sure that in the West End there were many people really anxious to help their fellow Londoners, though they might have very vague ideas as to how to set about it. He could confidently recommend this Society to their support; there was no doubt as to the good it did, and the objects aimed at were of immediate realisation in the increased comfort and health brought to the sick poor.

At the close of Lord Crewe's speech a collection was made, and the meeting concluded by the re-election of the Council and a vote of thanks to the Lord Mayor.

### Medico-Legal Society.

### MIDWIVES AND THE STATE.

A Meeting of the Medico-Legal Society was held on Tuesday at the rooms of the Royal Asiatic Society. The President (Mr. Justice Walton) took the chair.

Mr. J. Theodore Dodd, M.A., read a paper on the working of the Midwives Act, 1902, dealing with the great practical difficulty experienced by the certified midwife in regard to "sending for the doctor" when attending poor patients. According to the regulations, she was bound in all circumstances of danger or difficulty not to rely on her own skill, but to send for a medical man. What was she to do if the people could not pay? It was the duty of the medical officers of health to try and keep people alive and healthy, but it would be illegal for a Town Council to spend money in providing that children should be born alive and healthy. The Poor-law was in itself fairly satisfactory, the difficulty in practice being due to defective administration. In the first place the Local Government Board had said that no one was entitled to relief unless in a state of absolute destitution—a phrase hardly accurate, and certainly misleading. Then the Board and its inspectors had tried to induce Guardians to cut down medical relief, to make it a loan, or to use other deterrents. Besides this, the machinery for obtaining medical help was much too leisurely. By the official method if either the Union doctor or an emergency doctor attended a poor person without an order, he was not entitled to demand a fee. The Guardians might pay it, but they could only do so if they decided that the husband and wife were "destitute." Now, the people might be practically unable to pay, and the doctor quite unable to recover his fee, and yet the Guardians might think that the people were not "destitute," and so the doctor might lose his fee. Thus a direct application to a doctor might only cause extra delay, and in all ordinary cases the first thing to be done was to send to the relieving officer, for no individual Guardian could give an order. Accordingly the midwife would send the husband or some messenger to the relieving officer. Even if the latter was in his office, the inevitable enquiries took time; if he were out, the delay might be very great. The remedy he proposed was simple. If the people said they were unable to pay, let the request in writing of a certified midwife to a doctor to attend be equivalent to an order from a relieving officer, so that the Guardians should be bound in the first instance to pay the fee which they usually paid to the Union doctor. Then the Guardians would be at liberty to recover it from the responsible parties if they were able to pay. To afford this complete remedy legislation might be necessary, but a near approximation to it might be given by the Local Government Board and Guardians without waiting for an Act of Parliament.

Passing on to what he described as a problem of far greater importance, Mr. Dodd pointed out that competent women would not become midwives because they were not properly paid, and 1910 was approaching. Here again, if fully administered, the English Poor-law could afford a remedy. Whenever requisite, let certified midwives be part of the medical staff of the Union, their services to be available, free, under proper regulations, for those who could not afford to pay. Such an arrangement would not only reduce infant mortality, death and disease rates, but have an excellent effect on the poorer classes by introducing order and cleanliness into many houses. In Ireland it was generally admitted that no dispensary district ought to be left without competent midwifery attendance, in addition to that of the dispensing doctor. He had no objection, however, if it were thought better, to an arrangement by which the midwives should be part of the staff of the medical officers of health of the sanitary authority. The attitude of the Local Government Board and of the Guardians of the fossilised school made him feel that the lives of the women and children were not safe in their hands. Unless they could be speedily brought to understand that "women labouring of child" needed not only the prayers of the Church but the help of the State, he should be glad to see another body take their office and provide the "necessary relief" of which they had robbed the women and children so long.

A lengthy discussion disclosed some difference of opinion. Dr. Toogood criticised Mr. Dodd's proposals as tending to encourage thriftlessness and giving to an unqualified woman of a few months' training the powers of a Board of Guardians. Sir William Collins alluded to the primitive superstition which still characterised the lying-in room, and from which even his own profession had so lately emancipated itself. He agreed with the paper in its advocacy of the spread of knowledge among midwives and their patients. Among others who joined in the discussion were Miss Wilson and Miss Paget (members of the Central Midwives Board), Dr. Downes (of the Local Government Board), who deplored the friction which seemed to exist between midwives and doctors, Dr. Ambrose, Dr Gregg (inspector of midwives for Staffordshire), Dr. St. Aubyn Farrer (who considered that the difficulty of obtaining the doctor was exaggerated), and Dr. Wise.

The learned chairman, in summing up the discussion, pointed out the distinction between that "charity," whose claims Mr. Dodd had advanced in his reply, and the imposition on the ratepayer of the cost of providing for other people's wives and children. He expressed the view that no nation could be happy or prosperous which did not realise to the full its sense of parental responsibility.

### The Central Midwives Board.

A special meeting was held at Caxton House on Thursday, March 7, when seventeen midwives were cited to appear before the Board to answer the charges alleged against them. Dr. Champneys took the chair, and there were also present Miss Wilson, Dr. Dakin, Miss Paget, Mrs. Latter, Mr. Parker Young, and Mr. Bertram (solicitor for the Board). Only two women—Mary Pitt, of Staffordshire, and Sarah Morris, Cambridgeshire—appeared in person, and each was represented by a solicitor.

### The Use of the Thermometer.

Mary Pitt, No. 1,717, was called first, the accusations against her being :—

"That, being in attendance as a midwife at the confinement of Mrs. Johnson, of Cannock Road, Chadsmoor, on April 4, 1906, an dfollowing days, she was guilty of negligence in the following respects :—

"(1) The patient's temperature having risen above 100.4°, with quickening of the pulse on April 6, and so continuing on subsequent days, she did not decline to attend alone and advise that a registered medical practitioner be sent for, as required by Rule E 17 (*c*).

"(2) The patient suffering from hæmorrhage and from foul-smelling discharges during her attendance, she did not decline to attend alone and advise that a registered medical practitioner be sent for.

"(3) Habitual neglect to keep the register of cases, as required by Rule E."

Evidence was given by Mrs. Johnson, the patient, who said that her husband had three times asked Mrs. Pitt to send for a doctor, but she had refused, saying that it was not necessary. The midwife had assisted the patient to get up on the tenth day, though the hæmorrhage was still present. The patient continued to get up, the midwife looking in on her sometimes until the fourteenth day, when the husband sent for the doctor.

A written statement by the doctor was to the effect that he found Mrs. Johnson with a temperature of 104° and severe abdominal pain, suffering from septicæmia. The midwife had taken the temperature only once, on the day following delivery.

Dr. Gregg, Inspector of Midwives for Staffordshire, then gave evidence as to Mrs. Pitt's midwifery work in general, from which it appeared that the register was not properly kept, but that the midwife was fairly clean in person and appliances.

The Board came to the decision that the evidence against Mary Pitt was not sufficient to justify the withdrawal of her certificate, but cautioned her as to the proper use of the thermometer.

### More Cases from the Midlands.

Dr. Gregg also gave evidence in the cases of four other midwives, some of the charges being of a serious character. Eliza Gunter, of Birmingham, and Sarah London were accused of attending cases of puerperal fever and neglecting to disinfect themselves or their clothing and appliances before going on to other cases. The Board decided to strike the name of Eliza Gunter off the roll and cancel her certificate, and to remove the name of Sarah London by her own desire.

Elizabeth Forrest was charged with not wearing a dress of washing material, not possessing the requisite appliances and antiseptics, using a lubricant for her hands which she had been in the habit of using for children's sores, and neglecting to notify a case of puerperal fever; and it was alleged against Elizabeth Langston that she did not possess the requisite appliances and antiseptics, and neglected persistently to provide herself with them, and that she was not scrupulously clean and failed to keep her register. At their own request the names of both these midwives were removed from the register.

### Removed by Request.

Complaints of breach of rules were made in the case of the following midwives, and each was removed from the roll at her own request: Anna Hall, Worcestershire; Ellen Holmes, Worcestershire; Georgiana March, Portsmouth: Mary Muffitt, West Riding; Louisa Salmon, Essex; Maria Scofield, West Riding.

### Struck Off.

The following midwives were adjudged by the Board to be struck off the roll: Eliza Ashton, West Riding; Mary Ann Ashton, Oldham.

### Queen Victoria's Jubilee Institute for Nurses.

Miss M. M. White has been appointed superintendent of Lincolnshire Nursing Association; Miss F. Cotton has been appointed to Melbury, Miss A. Ellis to Gosport, Miss R. Griggs to Grantham, Miss S. H. Hodgson to Burnley, Miss A. Hoos to Sheffield, Miss Hilda Johnson to Leighton Buzzard, Miss C. Maskell to Radstock, Miss Henrietta Sykes to Goole, and Miss L. Steele to Wolboro'; Miss Alice Carlton has been transferred to Nottingham from Guildford; Miss Westcott to Northampton from Winsham; Miss Haddon to Gloucester, temporarily, from Milford Haven.

### Everybody's Opinion.

### A PROBATIONER LOSES HER SIGHT.

"Policy Nos. 5178 and 5175 Royal National Pension Fund for Nurses" writes: I send postal-order for 5s. for the nurse who lost her eyesight.

Miss M. J. Hayes, superintendent nursing sister, Queen Alexandra's Military Nursing Service of India, Umballa, Punjab, writes: I beg to forward postal order for 5s. for a nurse who lost her eyesight owing to an accident with an enteric fever patient.

### THE MIDWIVES ACT.

"North Country" writes: In the report of the annual meeting of the Association for Promoting the Training and Supply of Midwives, published in your columns, I read that in 1910 the midwives who are enrolled as *bona fide* will "go out," and therefore a great deficiency will exist in supplying their place. As I understand the Act such is not the case, and the *bona fide* midwife will be secure in her certificate after 1910, which will then, in my belief, create a slump in midwives. In this town every woman who ever stood by at a case of midwifery seems to have been placed on the roll, and some of the grossest kind. I suggest that this is an injustice to the trained and competent midwife who has paid for her training and is willing to work in the district that no distinction is made between the trained and the *bona fide* midwife. I find in going about amongst my patients the general impression is that they are all trained now, because, having a certificate, they must be competent.

### Appointments.

Banbury Infirmary.—Miss Evelyn A. Hall has been appointed charge nurse. She was trained at St. Olave's Infirmary, Rotherhithe, and has since done district nursing. She holds the certificate of the Central Midwives' Nurses.

Bedford County Hospital.—Miss I. I. Micklem has been appointed night sister, and Miss K. Chalker theatre sister. Miss Micklem was trained at Addenbrooke's Hospital, Cambridge, and holds the certificate of the Central Midwives Board. Miss Chalker was trained at the Norfolk and Norwich Hospital, and has since been sister of male wards at Stroud Hospital.

City Hospital for Infectious Diseases, Newcastle.—Miss Alice Blanche Booth has been appointed home sister. She was trained at the Northern Hospital, Liverpool, and has since been sister in charge of the Isolation Hospital, Lynn; night superintendent, home sister, and assistant housekeeper at the Northern Hospital, Liverpool; matron of the Isolation Hospital, Sheffield; and matron of the Cottage Hospital, Ramsbottom, Lancashire.

District Asylum, Ayr.—Miss Mary Christie has been appointed matron. She was trained at the Royal Infirmary, Aberdeen. She has since been day-and-night sister in Longmore Hospital, Edinburgh, and assistant-matron at Ayr District Asylum.

Egham Cottage Hospital.—Miss Constance M. Starling has been appointed matron She was trained at St. Thomas's Hospital, London, and has since been sister at the Bolingbroke Hospital, Wandsworth Common, S.W.

Fermanagh County Hospital.—Miss J. Stott and Miss S. Hinton Shaw have been appointed staff nurses. Miss Stott was trained at the Dumfries and Galloway Royal Infirmary, and has since been staff nurse at the Royal Victoria Hospital, Belfast. Miss Shaw was trained at Lewisham Infirmary, has been staff nurse at the North Eastern Hospital, London, and has done private nursing for the Richmond Home, Belfast.

Great Northern Central Hospital, London.—Miss Victoria Daunt has been appointed matron. She was trained at the Great Northern Central Hospital, where she was afterwards sister. She has since been night sister and acting matron of the National Hospital for the Paralysed and Epileptic, and matron of the London Homœopathic Hospital.

Kendray Hospital, Barnsley.—Miss B. Alcock has been appointed sister. She was trained at the Coventry and Warwickshire Hospital, Coventry, and has since been night superintendent at the Royal Hospital, Richmond, Surrey.

Long Grove Asylum, Epsom.—Miss Margaret Alison has been appointed matron. She was trained at Longmore Hospital, Edinburgh, and Western Infirmary, Glasgow. She has since been assistant-matron, Stirling District Asylum, Larbert, and matron of District Asylum, Ayr.

Nottingham Children's Hospital.—Miss Hutchison has been appointed staff nurse. She was trained and has been staff nurse at the Children's Hospital, Newcastle-on-Tyne.

Royal County Hospital, Ryde, Isle of Wight.—Miss Nora Letcher has been appointed sister. She was trained at Tunbridge Wells General Hospital, where she has since been holiday sister.

Sanatorium, Benenden, Kent.—Miss Annie Downes and Miss Muriel Candler have been appointed sisters. Miss Downes was trained at the Wandsworth and Clapham Union Infirmary, where she has since been charge nurse. She has since been head nurse and superintendent at Trowbridge and Melksham Infirmary, and night charge nurse at Frimley Sanatorium. Miss Candler was trained at the Royal Northern Hospital, Liverpool, and has since been in charge of a Mission Hospital at Mount Lebanon, Syria; sister at the Royal National Hospital, Ventnor; and sister at Mount Vernon Consumption Hospital, Hampstead. She has also done private nursing.

St. George's Infirmary, Fulham Road, London.—Miss E. A. Dexter and Miss E. A. Watkins have been appointed sisters. Miss Dexter was trained at the Lambeth Infirmary, and Miss Watkins at the Newport and County Hospital.

St. Marylebone Infirmary, North Kensington.—Miss S. J. Cockrell has been appointed matron. She was trained at St. Marylebone Infirmary, where she has since been staff nurse, ward sister, home sister, and assistant matron. She was subsequently lady superintendent of Birkenhead Infirmary.

St. Thomas's Union, Devon.—Miss Charlotte Annie Palmer has been appointed superintendent nurse. She was trained at Birmingham Poor-law Infirmary, and has since been nurse at one of the hospitals of the Metropolitan Asylums Board, and the Union Infirmary, Gloucester.

### Annual Sale at Andersons'.

(By our Shopping Correspondent.)

During the next fortnight until March 28 Messrs. Anderson, Anderson, and Anderson, of 37 Queen Victoria Street and 58 Charing Cross, are holding their annual stocktaking sale. Nurses will find that there are large reductions in every department. Ladies' long waterproofs are wonderful bargains, those costing £8 8s. being reduced to £2 2s. and others valued at 63s. being now only 21s. The shower-proof coats are reduced in some cases from 42s. to 25s., in others from 31s. 6d. to 10s. 6d. As to the surgical bargains, air-beds priced at 67s. 6d. can this month be purchased at a sovereign reduction; water-beds, originally priced at £7 10s., for £3 10s. and £4; and there is also a substantial reduction in cushions, sheeting, and enemas. A full and exhaustive catalogue can be obtained upon application, and orders can be sent through the post by those who are unable to purchase personally, and will be promptly attended to.

### A Book and its Story.

### LOVE AND REVENGE.*

Mr. Mitford's new novel is lacking neither in stirring incidents nor vivacity of treatment. Its theme is the old one of thwarted love and revenge, and the scene is laid mainly, as its title suggests, in Holland. The particular part of it sacred to the heroine is a little island in Friesland, "unrecorded on any map." Here she, an English girl, lives with her father, a particularly repulsive specimen of humanity, whose failing eyesight alone entitles him to the sympathy of the reader; and here she meets with the artist, Lewis Strangways, in search of isolation and the picturesque, who writes the story of the insane resolve of an injured man to haunt the rival who twenty years previously had married the woman who was his promised wife. Sir Samuel Portal, standing high in the medical world as the greatest oculist of the day, had attained celebrity suddenly after years of study and research, and had recently given to the world his discovery, which was to bring sight to those born blind, and by the same process to prolong the strength of vision into old age. He tells his story to Strangways when he meets him at his club in London. As a young man without means or influence he first met the woman who inspired him with the love of a lifetime, a love which neither desertion nor time could dim. Later mental breakdown, resulting from hopeless despair, and desire for vengeance on the man who had wrecked his happiness, followed. It is years since Strangways had met the doctor, but as a boy he had been a welcome visitor in his father's house, and Strangways, being now without parents or near relatives, was delighted to renew the acquaintance of one who had been the good fairy of his childhood. He looked with interest at the face of his friend. "As I watched him I realised what an exceedingly handsome man he was. His hair was nearly white, although he could not have reached his sixtieth year. His face was thin and oval in contour, and his features exquisitely formed. . . . His eyes, sunk deep under faintly pencilled brows, wore an expression that suggested many hours spent in solitude and at work. . . . From the moment when we first sat down till the time when we rose from the table his gaze wandered restlessly about the room, from one party of diners to another, searching even the remotest corners of the room through his pince-nez, and turning when each new arrival passed through the great glass doors. Was he looking for someone, or was this a natural optical condition of every great oculist? I wondered whether he had tried his great discovery upon himself. Perhaps he had, and this was the result of his treatment—a restlessness of eye that never seemed to tire." In relating the story of his life to Strangways, he dwelt with persistency on the desire for revenge far more than on the successful discovery which had made him famous.

He had come to that part of his story when, after leaving the asylum in which he had been confined for five years, he took up his life again. "I do not grudge the time, for it brought me rest. When I came out my brain was quite clear. I continued where I had left off. . . . Henceforth I live but for one purpose in life—I must find the man who stole from me the woman I loved." Strangways, seeing it is useless to discuss the subject with Sir Samuel, agrees, at his urgent request, to accompany him abroad for some months; and at the end of that time they part. After a brief stay in London, Strangways sets out for Friesland, and resumes his life as an artist. The morning after his arrival on the tiny island which he had chosen as his headquarters he opens his shutters early and looks out on to the quaint scene. "On either side of me stretched the toy Dutch fishing village, looking for all the world as if it had just been made in a doll shop and finished in a hurry. Sloping gabled roofs, green shuttered windows, crooked doorways of vivid blue or brown, lofty poplars standing like sentinels above the clustering cottages, with a hazy background of golden sand-dunes. . . . Far, far away upon the distant horizon I could see the brown sails of the dark fishing boats, dark against the limpid blue of the sky, looking like autumn leaves that were caught by a drifting tide. This was the fair scene that met my gaze. As I looked out across the waters I realised why it was that this north coast of Friesland was one of the most difficult to navigate in the world. The tide had receded far from the land, leaving a wide expanse of sand stretches with narrow channels between them, and the sun glowed red upon the miniature deserts which were scarcely hidden when the waters rose. . . . It must have required a clever pilot indeed to steer any boat through the darkness between those narrow, treacherous sand-banks." The scene was enlivened later when looking down the village street the school children were to be seen. "Their quaint, old-fashioned little figures were to be seen everywhere. They seem to have few games, and their occupations, like their costumes, resembled those of their parents as much as possible. Tiny creatures who could hardly walk paced seriously up and down the red-bricked dyke—most of the boys grouped in twos and threes, and all busy with their knitting." Unlike most of the Dutch communities, the entire population was Roman Catholic, and the island did not contain any Protestants. A charming picture of the parish priest, a real father to his flock, is given. He is standing in the room of his cottage that serves as sitting-room and study. "He made a beautiful picture as he stood there, his frail bent figure silhouetted against the evening light that sifted through the tendrils about the window. There was almost an inspired look upon his face as he turned to me. I have often since remembered that scene in the little barely-furnished room. The thin, stooping form, in its rusty cassock, and the glory of the setting sun behind him slanting through the window in a broad bar of golden sheen to the bare boards he trod. . . . He opened his hands, pressing the thin palms together as though he were offering up some silent benediction. 'I often think,' he said, 'these old hands of mine are like the binding of my books, for they include many pages of human life.' He continued in a dreamy whisper: 'With them I open the first chapter of many a life story as I sprinkle the holy water upon the face of infancy. With them I also close the last sad chapter when I give the mortal body back to the earth from which it came, and the soul returns to the good God Who gave it.'" Strangways gains from the old man some information about Izelle and her father. His first sight of her, as she comes up to him across the sands, and stands silently inspecting his canvas, is described. "The girl—she was little more than a child in years—smiled at me. . . . Then she paused, looking up and down my canvas with her pretty head turned slightly to one side and her brows arched in silent criticism. At that moment I knew that I had just looked into the most exquisite face I had ever seen or imagined." Mr. Mitford writes like an artist, and there is a charm about one part of his book as simple and compelling as the picturesque land and folk about which he writes. But the contrast between this portion and the lurid drama with which it closes is startlingly realistic.

* "Izelle of the Dunes." By C. Guise Mitford. (John Long. 6s.)

### Notes and Queries.

**REGULATIONS.**

**The Editor is always willing to answer in this column, withou any fee, all reasonable questions, as soon as possible.**

**But the following rules must be carefully observed.**

**1. Every communication must be accompanied by the name and address of the writer.**

**2. The question must always bear upon nursing, directly or indirectly.**

**If an answer is required by letter a fee of half-a-crown must be enclosed with the note containing the inquiry.**

*Corns.*

(231) Can you tell me of a remedy for corns? I have suffered much, but have found no remedy.—*Anxious.*

As the cause of corns is, in the majority of cases, pressure on soft parts by ill-fitting boots, the first thing to be done would be to make sure that the footgear is comfortable and easy fitting. Strict attendance to this prophylactic measure will probably prevent formation of corns. Those already existing may be treated by bathing the feet in hot water, rendering thereby the upper or horny layer of the skin soft; with a sharp corn knife this layer may then be shaved off over the corn, care being taken not to go deep so as to injure the true skin below, and draw blood. A corn-plaster, to be obtained from any druggist, may be placed over the corn thus treated. Steady perseverance in this course of treatment will probably effect a cure.

*Nursing Question.*

(232) Is it necessary when passing the rectal tube to place the other end in water, or is this only a matter of opinion?—*Fanny.*

Your question is not sufficiently explanatory. It seems to us that you should have very exact instructions from the medical attendant, as the process, for whatever purpose, should not be undertaken in ignorance, or much harm can be done.

*Nursing the "Hoppers."*

(233) Can you tell me where I can get information about joining a hopper camp as a nurse next season?—*A Hopper.*

If you write enclosing stamped envelope to the Rev. F. G. Oliphant, Teston Rectory, Maidstone, he will be able to help you.

*Lady Stewardess.*

(234) Will you tell me where to apply for a post as lady stewardess? I am a trained nurse.—*S. A. G., Italy.*

The only two companies who employ nurses as stewardesses are the Booth Steamship Company, Liverpool, and the Royal Mail, Southampton. But it is very rarely there are any vacancies.

*Canada and Australia.*

(235) Can you tell me where to apply for information? I want to get a hospital post either in Canada or Australia. I have had five years' general training.—*Chester.*

We fear you will find difficulty in getting a post, as both countries train as many nurses as they require, but the Victorian Order of Nurses for Canada, 578 Somerset Street, Ottawa, or the Royal Victorian Trained Nurses' Association, Melbourne, might kindly advise you.

*Poor-law Nurse.*

(236) If I train as a Poor-law nurse, shall I have any difficulty in getting a post at a large hospital?—*Thrale End.*

If you wish to devote yourself after your training to hospital work, it is wiser to train in a hospital.

*Incurable Home.*

(237) Do you know of any small homes for incurables, private and also paying patients? I want to go to such as a nurse. I am not trained, but have had experience in incurable illnesses.—*Snow Drop.*

You will find such a list as you require in "Medical Homes," price 6d., and "Homes and Hospitals for Gentlewomen," price 1s., published by the Army and Navy Stores. The Scientific Press, 28 and 29 Southampton Street, Strand, London, W.C., can obtain both for you.

**Handbooks for Nurses.**

| | Post Free. |
|---|---|
| "How to Become a Nurse: How and Where to Train." | 2s. 4d. |
| "Nursing: its Theory and Practice." (Lewis.) ... | 3s. 6d. |
| "Complete Handbook of Midwifery." (Watson.) ... | 6s. 4d. |
| "Preparation for Operation in Private Houses." ... | 0s. 6d. |
| "The Nurses' Enquire Within." ... ... ... ... | 2s. 3d. |
| "Nurses' Pronouncing Dictionary of Medical Terms" | 2s. 0d. |

Of all booksellers or of The Scientific Press, Limited, 28 & 29 Southampton Street, Strand, London, W.C.

### For Reading to the Sick.

### HEALING PAIN.

"Pain will bring thee joy at last,
When the pain is overpast."
"Yes, but pain is strong," she said;
"Pain will teach thee cunning lore,"
"The time is very long," she said.

Then thro' the dark there came a word;
A gentle thrilling Voice was heard:
"My daughter, it is I," He said.
"The Hand that weighs thee down is Mine;
The touch thou loathest is Divine:
Wilt thou not have Me by?" He said.

"Go not, my Lord—go not away."
Beneath the Hand she smiling lay.
"Here will I ever dwell," she said.
"The pain that makes Thy Presence here
Is healing pain, holy and dear;
Where Thou art, all is well," she said.

*Anon.*

Take it as the pledge that Jesus loves you, when, though the storm has continued to rage, and the calm has been delayed, the waves have not been allowed to overwhelm you. His time is the best time. Yet a little while, and the hour of deliverance will come. Yet a little while and you will have rest and peace and quiet. You will find that it was good for you to have been afflicted—that your faith was strengthened by trial—that your progress heavenward, instead of being retarded, was hastened by the storm—that the winds you dreaded were wafting you onward in your voyage—and that the waves which seemed to threaten you with death were bearing you to the Haven of eternal calm. O then, whatever be your present state—whatever the cares and troubles and griefs which burden your spirit—whatever the darkness which has been permitted to enwrap you—strive ever to feel that He, Who has for a season seemed to leave you all alone on a stormy sea—He Who has spoken to the tempest, and allowed the waves to rear their foaming crests—is even now pleading for you on the mount, even now watching you, till the hour arrive when He shall say "Peace, be still!" and drawing near to you, shall whisper these consoling words, "It is I: be not afraid."—*Able to Save.*

When sorrow comes to you, or to those you love, do not shrink from it as from some cruel torture, but welcome in it an angel to bring you near to God. Do not fear—do not fear. "This light affliction, which is but for a moment, worketh for us a far more exceeding and eternal weight of glory." Our God is enough for every one of us; and when we pass behind the cloud, it is that we may see His Face, and our joy may be full.—*Bishop Thorold.*

### PRAYER.

Almighty and Merciful God, Who art the Strength of the weak, the Refreshment of the weary, the Comfort of the sad, the Life of the dying, the God of patience and of all consolation, help me, O Eternal and Pitying God, help me to possess my soul in patience to maintain unshaken hope in Thee, and to keep that childlike trust which feels a Father's heart hidden beneath the cross.—*T. Havermann*, 1516.

## Figures and Tables

**Figure f1:**